# Availability, Toxicology and Medical Significance of Antimony

**DOI:** 10.3390/ijerph19084669

**Published:** 2022-04-12

**Authors:** Argyrios Periferakis, Ana Caruntu, Aristodemos-Theodoros Periferakis, Andreea-Elena Scheau, Ioana Anca Badarau, Constantin Caruntu, Cristian Scheau

**Affiliations:** 1 Department of Physiology, The “Carol Davila” University of Medicine and Pharmacy, 050474 Bucharest, Romania; argyrios.periferakis0920@stud.umfcd.ro (A.P.); theodoros.periferakis0920@stud.umfcd.ro (A.-T.P.); anca.badarau@umfcd.ro (I.A.B.); costin.caruntu@gmail.com (C.C.); 2 Akadimia of Ancient Greek and Traditional Chinese Medicine, 16675 Athens, Greece; 3 Department of Oral and Maxillofacial Surgery, The “Carol Davila” Central Military Emergency Hospital, 010825 Bucharest, Romania; 4 Department of Oral and Maxillofacial Surgery, Faculty of Dental Medicine, “Titu Maiorescu” University, 031593 Bucharest, Romania; 5 Department of Radiology and Medical Imaging, Fundeni Clinical Institute, 022328 Bucharest, Romania; andreea.ghergus@gmail.com; 6 Department of Dermatology, Prof. N.C. Paulescu National Institute of Diabetes, Nutrition and Metabolic Diseases, 011233 Bucharest, Romania

**Keywords:** antimony, stibnite, toxicity, exposure, health impact, pathophysiology, resistance

## Abstract

Antimony has been known and used since ancient times, but its applications have increased significantly during the last two centuries. Aside from its few medical applications, it also has industrial applications, acting as a flame retardant and a catalyst. Geologically, native antimony is rare, and it is mostly found in sulfide ores. The main ore minerals of antimony are antimonite and jamesonite. The extensive mining and use of antimony have led to its introduction into the biosphere, where it can be hazardous, depending on its bioavailability and absorption. Detailed studies exist both from active and abandoned mining sites, and from urban settings, which document the environmental impact of antimony pollution and its impact on human physiology. Despite its evident and pronounced toxicity, it has also been used in some drugs, initially tartar emetics and subsequently antimonials. The latter are used to treat tropical diseases and their therapeutic potential for leishmaniasis means that they will not be soon phased out, despite the fact the antimonial resistance is beginning to be documented. The mechanisms by which antimony is introduced into human cells and subsequently excreted are still the subject of research; their elucidation will enable us to better understand antimony toxicity and, hopefully, to improve the nature and delivery method of antimonial drugs.

## 1. Introduction

Antimony (Sb), as an element, has been known since ancient times and has been used by many civilizations for different purposes. It is classified as a heavy metal, since it has a specific density of more than 5 gr/cm^3^ [1], and it has adverse effects on the health and physiology of living organisms [2]. Heavy metals such as antimony are released into the biosphere mostly via weathering and erosion, industrial and mining activities, and pest control agents [3]. Since antimony belongs to Group 15 of the periodic table, it is also referred to as a metalloid [4].

As a metal, antimony is not affected by humid air and pure water, but if melted by temperature, it ignites. It is known to react violently with elements of the halide group (F, Cl, Br, and I) thus forming trihalides. The detailed chemistry and properties of different phases of antimony are described in detail in [5].

Antimony has been known since antiquity in China and in Egypt, and there is proof that even the Chaldeans knew how to sequester it from other ores [6]. Accounts from Greek and Roman authors of the era refer to its main use as an eye ointment for its cosmetic and medicinal properties [5]. Around the 16th century, it was realized that antimony was useful in separating gold from silver. However, until the dawn of the 20th century, the demand for and use of antimony remained fairly low [7]. It would be during the First World War [8], and subsequently, during the Second World War, that demand for antimony increased, as it did for other materials such as oil [9] and emery [10,11,12]. After the 1950s, the dominant market of antimony became the plastics industry, which currently consumes over 60% of the produced antimony, both as a catalyst and a flame retardant. The pyrometallurgical, hydrometallurgical, electrometallurgical, and mineral processing processes currently employed at an industrial level are detailed in [13].

The usage of antimony derives from its particular properties: it is hard, brittle, and non-malleable, and this renders it unsuitable for being used in the same manner as other metals, such as Pb, Fe, etc. Instead, it is used in small amounts in alloys to enhance their strength and hardness, and it may be also used ornamentally. In the electronics industry, its use for diodes is increasing [13]. The basic artificial industrial compounds are antimony trioxide (Sb_2_O_3_), antimony pentoxide (Sb_2_O_5_), sodium antimonate (NaSbO_3_), antimony trisulfide (Sb_2_S_3_), antimony pentasulfide (Sb_2_S_5_), and antimony triacetate [Sb(CH_3_COOH)_3_]. They are mostly used as flame retardants and catalysts (Table 1).

Recently, antimony was included in the critical raw materials (CRM) list. These are materials that are characterized by increased economic importance, high-risk supply chains, and the inability of substitution by materials of commensurate properties [14]. According to [15], the approximate amount of antimony exceeds 1.5 × 10^6^ t worldwide. As of 2019, the worldwide reserves of antimony range between 50,000 t, in Tajikistan and 480,000 t, in China, with significant reserves being present in Australia (14,000 t), Bolivia (310,000 t), Mexico (18,000 t), Pakistan (26,000 t), Russia (350,000 t), and Turkey (10,000 t).

Antimony is currently virtually non-recyclable [16], due to a number of reasons [17,18], with only a few successful recycling examples existing [19], thus making it a critical material. In the beginning of the 20th century, the major suppliers of Sb were Bolivia and China, later joined by South Africa and the U.S.S.R. [6]. After the 1980s, China showed a rapid and sustained expansion of Sb mining enterprises, and is now responsible for about 87% of the global production [20].

Both heavy metals and metalloids present a particular danger to human health, since they are not biodegradable, and are therefore prone to accumulation in biological systems [21]. A particular issue with heavy metals is that, in contrast to other toxins, they are not destroyed but rather recycled constantly through the geosphere and the biosphere [22,23,24].

Antimony toxicology is relatively well documented, owing to its presence in urban environments, its use in a small number of drugs and the relative studies on occupational exposure. In fact, the atmospheric Sb compounds from anthropogenic sources are steadily increasing [25,26,27], thus predisposing a significant part of the population to potentially associated pathologies.

In this review, we will concisely examine the geological processes associated with antimony ore deposits and the basic characteristics of important antimony minerals. Subsequently, we will present the current research on exposure to antimony from an environmental and an anthropogenic standpoint, both at mining sites and in urban settings. We will then examine the toxicology of antimony, the associated pathologies in every major physiological system, and the relevant mechanisms of pathogenesis. Finally, we will go through the use of antimony in medicine, more specifically in the treatment of tropical diseases, and discuss briefly the emerging problem of antimonial resistance.

## 2. Mineralogy, Geochemistry, and Availability of Antimony

It is necessary to understand the geological properties of antimony in order to properly assess the impact of related geological processes on public health [28,29]. Antimony exists naturally in the Earth’s crust and is released into the environment via both natural and mining–industrial processes. Based on current research [30], Sb levels are below 1 mg/kg in rocks and soils, and 0.1 mg/kg in flora and waters. It exists in two biologically relevant oxidation states [31]: the pentavalent form of antimony is common under aerobic conditions, and the trivalent form is common under anaerobic conditions. The availability of antimony in the environment can be regulated by a host of different technological processes, including but not limited to, coagulation, membrane separation, ion exchange, adsorption, and phytoremediation, as presented in [32].

The most common pentavalent-type Sb is found in seawater and freshwater, while antimony released from anthropogenic activities will be most commonly trivalent. The speciation of antimony, from Sb(III) to Sb(V) has already been documented by [33,34]. In the pentavalent state, antimony is very stable and forms complexes with numerous ligands [35,36]; conversely, its trivalent form is a weak base of electropositive character [37].

Antimony itself is a white lustrous metal of average hardness. Geochemically, it is classified as a chalcophile element, meaning that it occurs along with sulfur and Cu, Pb and Ag [13]. There exist over 100 minerals containing antimony [38], belonging to various mineral classes; only a few, however, are important from an economic standpoint (Table 2).

The principal antimony ore exists in the form of stibnite (Sb_2_O_3_), a sulfide mineral macroscopically appearing as columnar or needle-shaped crystals. The color of the crystals is most commonly a silvery to dark grey, although tarnished crystal faces may have an indigo blue coloration. Stibnite forms in hydrothermal systems and is associated with cinnabar, quartz, and fluorite [55]. Antimony occurs in a number of different deposits such as Sb-bearing minerals (boulangerite, a lead-rich mineral), bournonite [56], gudmundite (an iron-rich mineral), and polybasite, which have also been recognized as minor antimony sources [57]. Antimony ore is frequently associated with undesirable elements, such as Hg [58]. Other accessory minerals are also associated with antimony ores, as mentioned in [57].

According to [57], antimony ores are associated with a number of different deposits, namely epithermal deposits, pegmatite deposits, and hot spring-related replacement deposits. Depending on the ore grade, antimony ore deposits can be categorized as either primary or secondary; in this second category, Sb is mined as a by-product. Sb deposits may be simple (Sb-rich) or complex polymetallic [59]. They can form in many geological settings, and examples of Sb deposits and prospects can be found in the literature [60,61,62,63,64,65,66,67,68,69,70,71,72,73]. A detailed account of the currently active Sb deposits is available in [56].

## 3. Exposure to Antimony

Exposure to antimony is more frequent in industrial and mining settings but it is also possible during everyday life. At any rate, the recommended maximum exposure level to antimony—also abbreviated as total daily intake (TDI)—is 0.6 μg per kg of body weight per day, as proposed by the WHO [74]. More detailed results, describing different natural and anthropogenic Sb levels, are included in recent research [75].

Extensive use of Sb caused by rapid industrialization and urbanization of the environment rapidly transformed the geochemical character of the soil of many areas [76], mainly due to indirect and direct pollution. A further compounding factor is that the content of such metal pollutants in the soil remains generally elevated, even decades after the removal of the polluting factor, since metals and metalloids exhibit prolonged soil residence times, as described in [77]. In the atmosphere, Sb also presents a potential danger in certain settings; atmospheric Sb compounds are attributable to waste incineration, the use of fossil fuels in internal combustion engines, and road traffic [78,79]. In this part, we will present the contamination and exposure at the different levels, where Sb is introduced into the biosphere due to anthropogenic activities.

### 3.1. Environmental Contamination and Exposure at Industrial, Mining, and Urban Settings

In appreciating the degree of land contamination, it is important to quantify the degree of uncertainty in the methods used to assess soil contamination. A suitable model is that of a probabilistic determination which allows for the determination of an uncertainty factor, as proposed in [80].

As mentioned, industrialization and urbanization triggered a rapid increase in heavy metal content in the biosphere. Soil is regarded as a major reservoir for potentially toxic elements [81]. In general, regarding pollutants, and in particular, antimony, the geochemical character of the soil greatly affects soil chemistry and therefore pollution levels; relevant examples are described in [82,83]. The importance of the geochemical profile of the soil cannot be understated. For example, in a study examining the pollution around the area of Lavrion, Greece, it was found that the availability of toxic elements was limited due to their sequestration in stable mineral phases [84]. This is very important, due to the extensive mining of the carbonate-replacement ore deposits of Lavrion [85], both in antiquity [86,87] and in modern times [88]. If, after aggregate centuries of mining, it is possible for the soil to ‘absorb’ some of the pollutants, then this creates novel opportunities and challenges in assessing industrial heavy metal contamination in general, and Sb contamination in particular.

Mining areas are particularly polluted with heavy metals, and only a small portion of heavy metal content can be attributed to geological processes, notably weathering and pedogenesis [84]. Air quality tests indicate that, while some areas with active mining enterprises enjoy reasonable air quality (e.g., [89]), in the majority of cases air quality is negatively affected. In fact, the environmental pollution, which is attributable to mining enterprises, has already been documented by numerous researchers for both ancient (e.g., [90]) and modern mining sites (e.g., [91,92,93,94,95]).

Most of the antimony will enter the environment due to mining and industrial activities [96,97,98]. Indeed, the levels of antimony around smelter sites are exceedingly high, a fact already noted in [99,100,101]. A particular role is played by waste incineration [102,103] and internal combustion engines. The use of fossil fuels aggravates the situation, given that coal typically contains Sb [103,104]. In contrast to the reference levels provided by [30], in mining areas, soil Sb concentrations can be over 2 × 10^3^ mg/kg and groundwater concentrations over 6 × 10^3^ μg/L. Plant levels may reach up to 143.69 mg/kg [105].

China’s largest deposits are the Xikuangshan Sb ore field, the Dachang Sn-Pb-Zn-Sb ore field, the Zhazaixi Sb deposit, the Xiangxi Au-Sb-W deposit, but of course, many others produce considerable amounts of Sb every year. Extensive research has been performed all these years in most of the mining sites of China (see [106] and references therein), and the results showed significantly increased Sb contents in the soil, water, and plants of most of the mining areas; more specifically, ref. [107] mentions that in the Xikuangshan area, Sb concentrations in the water exceed 53.6 μg/L. Similar examples of Sb contamination are recorded from mining sites in Massif Central, France [108]; Dúbrava, Slovakia [109]; Su Sergiu, Sardinia, Italy [110]; Glendinning, Scotland [111]; Endeavour Inlet, New Zealand [112]; Barcelona [113] and Zamora [114], Spain; Keramos, Chios Island, Greece [115]; and multiple sites in Poland [116,117,118]. Of course, this is only a small fraction of the recorded cases, and given the current research trend, more papers on the subject will surely come up. Antimony values, in soil and water samples for some of the mining areas referred to above are provided in Table 3.

Regarding the occupational exposure to antimony, a notable problem, concerning its quantification, is that it frequently coexists with other toxic elements, such as As and Pb. As such, it may be difficult or even impossible to separate between the different toxicities [120]. Be that as it may, if protection standards are maintained, save in cases of accidents, the danger is minimal; in countries where, for a host of reasons, safety regulations are lax [121], the danger is increased.

Antimony also accumulates in plants, where it enters, at a cellular level, using a number of different aquaporins [122,123]. It is known that there exist numerous Sb species, both stable and unstable, in aqueous environments [25,124,125]. The trivalent form will be predominant in reducing to mildly reducing conditions [126]. The pentavalent form exists in oxidizing environments, such as soils [127,128,129]. However, Sb(III) prevalence—this is the most toxic species—was recorded in a few cases in [130,131]. In any case, direct human contact with contaminated soil is one of the major exposure pathways, as outlined in [129]. In general, antimony bioavailability in plant-dominated environments exhibits notable variability, e.g., [100,132,133,134,135,136,137,138]. Even though antimony is non-essential to plants, it can be taken up through their roots if and when it is available in water-soluble forms [101]. Some plant species, notably *Achillea ageratum*, *Plantago lanceolata*, and *Silene vulgaris*, accumulate antimony readily [101]. For example, according to the results of the aforementioned research, plant concentration can exceed 440 ppm, depending on the plant species and the uptake mechanism. At any rate, Sb accumulation in plants remains pronounced around mining areas [79,139].

In urban settings, a study from Athens, Greece [140], indicated that As is prominently accumulated in parks and woodland areas within the city; taking into account that inorganic As and Sb exhibit similar chemical behavior, it is possible that further research will reveal Sb enrichment in these areas. More prominently, Sb is released into the air by the burning of fire retardants [141], and by brake abrasion particles [142], released during car braking. Recent research has revealed that Sb serum concentrations are elevated in children younger than 6 years in age, in Bucharest [143]. Given the fact that as far as European cities go, there are far more polluted ones, further research is required to establish the susceptibility of young children to heavy metals in urban settings.

### 3.2. Exposure to Antimony Related to Water Consumption

The first obvious source of antimony intoxication would be through the tap water, but the concentration of antimony is usually well below the accepted limit of 1 μg/L [25]; exceptions to this fact are some isolated reports from environmental agencies [144]. A large part of Sb in drinking water is eliminated via water sanitation methods, the most efficient and cost-effective of which is coagulation–flocculation and adsorption [145]. Other methods for achieving the same purpose have also been proposed [146,147,148,149]. Despite the efficacy of such methods, the presence of natural organic matter in the water increases the risk of human exposure [150], a fact already reported by [147,151]; the total carbon content is an additional negative modifier for Sb clearance from potable water [152]. The negative influence of natural organic matter can be explained by the formation of Sb–organic matter complexes [153]. Sb binds preferentially with hydrophobic ligands, rather than hydrophilic ones, and the binding potential is higher for Sb(V), compared to Sb(III); the presence of Fe reduces this potential [150].

The dominant species is the pentavalent form of antimony, as reported by [154]. According to the study of [144], the predominance of Sb(V) is explained by the oxidizing agents used during potable water processing, and by the limited stability of Sb(III) in aqueous solutions.

Despite reports of no Sb contamination in the overwhelming majority of tap water supply, the situation is rather different for bottled water. In the plastics industry, antimony trioxide (Sb_2_O_3_) is used as a catalyst in polyethylene terephthalate production (PET); consequently, the plastic making up the bottles contains between 190–300 mg·kg^−1^. Due to antimony leaching, the amount of antimony in the water is directly proportional to the duration of plastic water bottle storage [155]. This problem is further aggravated by the increase in bottled water consumption during the last decade [156,157,158,159,160]. The storage of plastic water bottles at higher temperatures increases antimony leaching, further contaminating the water contained within [155,161,162,163].

### 3.3. Exposure to Antimony Related to Food Consumption

The entry point of Sb into the food chain is through plants, which absorb it from contaminated soil. However, the degree of antimony soil contamination is not the sole determining factor, as its mobilization in the soil greatly affects its uptake by the local flora; this has been demonstrated in [134,139]. Another modifying factor is the position of the plants in the food chain, i.e., if the plant is consumed directly by humans, or if it enters the food chain after being consumed by herbivores. This was demonstrated in [164,165,166] for mushrooms and radishes.

Some data on the presence of Sb in milk exist [167,168,169,170]. The intake of antimony from milk was calculated to be less than the limit applicable in the case of water, but the comparison between the results of different studies is sometimes difficult, due to different calculation methods. Studies on wine samples indicate that most often antimony is below the detection limits of the applied methods; the use of other methods indicated Sb levels close to 10 μg·L^−1^ in some European wines [170].

Seafood is generally considered not to be a source of contamination in most areas, although data from industrial coastal zones indicate that this is not always the case [171]. It is believed that industrial activity is the direct cause for the results of this study. The predominant species of antimony in seafood is the pentavalent form.

But even if food itself is not considered a high-risk source for Sb, its packaging is not exempt as a source for concern. The plastics used in food packaging are manufactured by the same process used for water bottle manufacturing and are therefore prone to contaminating packaged food with antimony, an occurrence which becomes especially pronounced if the plastic container is heated in a microwave oven [144]. Research by [172] also indicates that the presence of citrus juice may increase antimony leakage from the plastic packaging; it is hypothesized that the citric acid preserves the oxidation state of the leached Sb(III), a process already demonstrated in [173]. Even in the latter case, however, the antimony content of beverages was below the acceptable levels.

## 4. Toxicity and Toxicology of Antimony

In general, heavy metal toxicity affects negatively the body’s systems, and long-term exposure will lead to the appearance of degenerative phenomena. The toxicity of antimony is monitored by assessing various environmental factors [174]. In addition, not all Sb which enters the body will participate in adverse reactions. Rather, only a fraction of it, attached to water molecules or various particles, will enter through the respiratory and/or the gastrointestinal tract [144]. The current trend in assessing human exposure to pollutants has shifted towards calculating the bioaccessibility of each pollutant and not just its total content [175,176,177]. An example of such an application was illustrated for Pb by [178].

The toxicity of antimony is regarded to be on par with, or even higher than, that of arsenic, and the inorganic form of antimony, Sb(III), is far more toxic, than the organic one, Sb(V). In general the inorganic species are most often the more potent forms in terms of toxicity. Despite antimony’s similarities to arsenic, it should be noted that only the biochemical behaviors of the trivalent form are comparable [168]; the pentavalent forms of antimony and arsenic have different structures [179] and may thus affect different physiological mechanisms of the human body [4].

According to [180], the toxicity of antimony is derived by its binding to thiol-containing enzymes. Specifically, the organic form of antimony is almost harmless to red blood cells, since it cannot penetrate their cell membrane, while the inorganic form shows a high affinity both for red blood cells and thiol groups. As antimony complexes with thiol groups, forming thioantimonites, it is presumed that the GSH levels within the cells are depleted, an effect already observed during exposure of cells to As [181], which exhibits comparable chemistry and toxicity, and also complexes with thiol groups. It is also not improbable that the thiol groups of some proteins interact with Sb in a similar manner to the thiol groups of glutathione.

Glutathione peroxidase is also affected negatively by Sb, and this decreases free GSH levels, leaving the cells yet more susceptible to oxidative stress [182]. Sb and As are direct inhibitors of pyruvate dehydrogenase, the basic regulatory enzyme determining the mode of glucose oxidation, i.e., anaerobic or aerobic. Exposure of cells to antimony leads to an observed drop in ATP levels, and it is hypothesized that the inhibition of pyruvate dehydrogenase by Sb, activates the anaerobic glycolysis pathway. Anaerobic glycolysis is, of course, vastly more inefficient than aerobic glycolysis and produces far less ATP. It is also hypothesized that the trivalent form of antimony is potentially carcinogenic [4]; currently, it is regarded as being carcinogenic for animals [183].

Up until recently, the mutagenic and carcinogenic potential of antimony had received meager attention, compared to studies on other heavy metals such as Pb, e.g., [184,185,186,187]. A recent study [187] ascertained that there is a probable correlation between the level of antimony trioxide inhalation in Sb smelter workers and detected DNA lesions. The mechanisms associated with Sb-mediated DNA damage are discussed in [188,189] and presented in a concise way in [75]. It is interesting to note that DNA damage was analogous to the urinary antimony levels; this study concurs with the findings of the only other significant study of Sb genotoxicity, [190]. Some researchers have performed animal trials, to determine if Sb is genotoxic, but the results were either negative [191] or marginally positive; thus, there is as of yet no consensus in the scientific community. Nonetheless, in vitro experiments in cells proved that Sb can cause cell death [192], inhibit DNA repair mechanisms [193], and interfere with transcription mechanisms [194]. Currently, the prevailing hypothesis is that Sb genotoxicity is mostly caused by its interference with repair mechanisms [195,196].

Antimony also interferes with the metabolism of sugars in the human body and can bind to many of them [197]. Its trivalent form inhibits gluconeogenesis [75] and promotes the pentose phosphate pathway [198]. Imbalances in lipid metabolism, caused by Sb, may also enhance its carcinogenic potential [199,200]. Sb(III) has been also linked to a hemolytic mechanism by [201].

Regarding the effects of Sb on the reproductive capacity of humans, there are as of yet no definitive conclusions. At first glance, the most severe effects seem to be associated with the increased mutation rates caused by Sb-related DNA damage leading to abnormal genotypes in offspring. According to [202], Sb exposure is linked to decreased sperm count, although based on relevant research [203], there is no decrease in semen quality, at least when Sb concentrations in the plasma are low. Moreover, in pregnant women, Sb accumulation is linked to increased incidence of pregnancy-induced diabetes mellitus [204,205] and perhaps hypertension [206]. Other pregnancy-related and development-related risks are described in [75].

### 4.1. Cellular Mechanisms Associated with Antimony Entry and Processing

Antimony enters the cells via aquaporin channels [207,208,209]. More specifically, it has been proven that Sb(III) enters the cells through the GlpF aquaporin channel in *Escherichia coli*, the same channel that mediates the entry of As(III) into that organism [210,211]. The GlpF protein belongs to the sub-family of aquaglyceroporins, because it allows not only water, but small uncharged solutes to pass through [212,213]. Later [213] proved that the Fps1 protein of the same sub-family is responsible for the entry of Sb(III) in *Saccharomyces cerevisiae*, thus illustrating the entry pathway in a eukaryotic cell for the first time. Because these proteins exist in cells of all gena and species, it can be said with a measure of certainty that this is the evolutionarily conserved pathway for the entry of metalloids into cells [207,208,209]. For humans, AQP9 is implicated in antimony transport, according to recent experiments [214,215]. Given the bidirectionality of aquaporins [122,216,217], it is probable that they may transport Sb out of the cell too, thus acting also as a detoxification mechanism. Based on the fact that the GLUT1 transporter [218,219] and hexose permeases can catalyze As(III) transport [197] in some non-human cells, it can be hypothesized that such proteins might be implicated on Sb(III) transport as well. The route of entry of Sb(V) remains unknown [4]; perhaps a clue may lie in the phosphate transporters which have been shown to transport As(V) [208,220,221], but this is a contested issue given the differences in the biochemical character of this particular oxidation state of these two elements.

Regarding Sb reduction intracellularly, the only Sb-specific mechanisms are known from unicellular organisms of the *Leishmania* species [222,223,224,225]; potentially some correlation to human cells can be made in the future. A more general mechanism, which was initially hypothesized as one of the causes of toxicity by [182] is the interaction between Sb and glutathione, as analyzed in [222].

### 4.2. Physiological Mechanisms of Sb Toxicity Reduction in the Human Body

As mentioned above, antimony is potentially carcinogenic, but some of its toxic potential is reduced by a host of cellular mechanisms, which decrease its cytosolic content. Cells can limit Sb import, force its export, sequester it in intracellular organelles, or possibly chelate it [226,227,228,229]. It has been proposed that the rapid expulsion of antimony from the cells might be related to the development of resistance to it.

The reduction of antimony from its pentavalent to its trivalent form is the principal mechanism behind the action of antimonials against leishmaniasis [230]; this will inhibit the action of glutathione and trypanothione. It can potentially then be expelled via the As pump [231].

Recent research [222] indicates that the reduction of antimony toxicity is regulated by the availability of glutathione. Glutathione catalyzes, via a redox reaction, the chemical reduction of Sb(V) to Sb(III), in a dose-dependent manner. This reaction happens faster in acidic pH values and at higher temperatures. Based on a similar redox reaction between glutathione and As [232], it is reasonable to assume the creation of an SbGS_3_ complex, a probability further supported by the findings of [233]. The total oxidation reaction may be written thus:

(1) $${\mathrm{SbO}}_{3}^{-}+H^{+}+2\mathrm{GSH}↔{\mathrm{HSbO}}_{2}+\mathrm{GS}-\mathrm{SG}+H_{2}O$$

where GS-SG represents the oxidized form of glutathione. The thermodynamical parameters of this reaction are presented in [234].

Antimony is expelled by the human body through renal filtration and excretion in urine [235], with different excretion rates recorded in China and Sweden by [236,237], respectively. Methylation, both by human cells and gut microbiota, has also been linked to Sb neutralization and removal from the human body [238,239].

### 4.3. Effects on the Respiratory System

There is an incomplete set of data, regarding the absorption of antimony in the respiratory tract. It has been established as a quantifiable occurrence, both from the studies on occupational exposure to antimony and relevant animal experiments. According to [240], the average absorption is 15%, a percentage similar to what occurs in the gastrointestinal tract. Particle size and solubility were considered as the main modifying factors.

Several researchers [241,242,243,244,245,246] have recorded elevated Sb levels in the blood and urine of workers exposed to antimony in mining and industrial settings. Given that the only form of exposure in these studies involved inhalation, it is evident that at least some degree of absorption must happen in the respiratory tract. Another research by [247], specifically on pregnant women working in Sb smelters, revealed that Sb was detectable in the placenta and the amniotic fluids. A confounding issue was that the levels of Sb in body tissues and fluids were not enough evidence to quantify absorption, and the chance that a portion of the inhaled antimony had been ingested and thus removed from the respiratory tract altogether before absorption, could not be excluded [144].

The systematic research of [248,249,250,251] proved that concentrations of Sb were elevated in the lungs of occupationally exposed people, thus corroborating that inhalation is proportional to some degree to the Sb air particle content. Studies on animals have also been performed [241,252,253,254,255,256,257,258]. But these suffer from more or less the same constraints presented below for animal examples on antimony absorption in the gastrointestinal tract. This question had already been raised in [259]. Finally, painless ulceration and perforation of the nasal septum was described in [260,261] in occupationally exposed workers. Even though Sb may be the culprit, it is hypothesized that the coexistence of As in these settings is the most probable cause [262].

### 4.4. Effects on the Cardiovascular System

It has long been recognized that the exposure of mammals to Sb-containing compounds is particularly dangerous for the cells of the myocardium. The first indication of this phenomenon was [263], in experiments with rats. Further research [241] indicated that the administration of potassium antimonyl tartrate increased the degeneration of the fibrous and connective tissue of the heart, even at low doses.

The adverse effects of exposure to antimony were also studied in the exams and autopsies performed on patients who had received antimonial drugs; Sb was identified as being the cause of death, due to its specific toxicity to the heart, an effect observable even in the altered form of some electrocardiograms [264,265,266]. The earlier study of [241] had correlated Sb-induced heart problems, also detectable by abnormal electrocardiograms, with the death of workers exposed to antimony trisulfide for a relatively prolonged average period.

It has been already proven that antimony increases the oxidative stress in myocardial cells [267], and the subsequent experiments of [182] proved that in vitro, myocardial cells exposed to Sb exhibited increased cell death incidence. This is tied to the presumed decrease in GSH levels and the interdiction of Sb in the activity of certain enzymes, as described above. The death of myocardial cells must also be associated with the observed drop in ATP levels, in cells exposed to Sb [182].

### 4.5. Effects on the Oral Cavity

Antimony is among a variety of metals that can be detected in the oral cavity and may be introduced as part of various dental materials [268]. A recent study using photoactivation analysis found trace elements of antimony alongside nickel, barium, arsenic, strontium, and others in dental composites manufactured by various producers [269].

The long-term release of antimony from dental materials might cause chronic exposure with severe effects. A study [270] using cell viability assays has shown that antimony demonstrates weak embryotoxicity, a finding that correlates with previous similar reports [271,272]. Furthermore, the presence of antimony in the oral cavity can induce a change in the salivary microbiome composition. In their recent study, the authors of [273] proposed that the presence of salivary metals will induce changes in the oral microbiome and lead to oral health issues. In subjects with increased antimony levels, they reported a higher abundance of *Lactobacillus* and *Granulicatella* species, which have been associated with the development of dental caries and increased dental decay [274,275].

Conversely, a recent study concluded that electronic cigarette smoking is not a source of increased antimony levels in the body, as the tested urine of regular e-cigarettes smokers showed similar levels of antimony and other heavy metals to that of persons who never used electronic cigarettes [276].

### 4.6. Effects on the Gastrointestinal Tract

The absorption of antimony through the gastrointestinal tract is estimated to be about 5–15% of the total amount of Sb ingested [240,277,278].

It has been established that antimony causes notable side effects when introduced to the gastrointestinal tract. Its use as an emetic has already been mentioned. There are some cases of oral poisoning reported [246,279,280] which indicate, firstly, that up to a point, Sb is absorbed in the gastrointestinal tract, and secondly, that it is poisonous even if we accept that the maximum absorption is only 15% of the ingested value. A single case study [246] reported that antimony was not detectable in the gastric juice and bile after 100 h, but serum and urine levels remained abnormally high even after one week. There have been numerous in vivo studies in animals [255,281,282,283,284,285] that have yielded a maximum of 18% gastrointestinal absorption of antimony tartrate. It was observed, however, that there were significant differences regarding absorption based on the delivery method. Intraperitoneal injection of the drug proved lethal whereas the oral administration was not, due to the poor gastrointestinal absorption of antimony [285]. Lastly, the authors of [286] conducted experiments regarding the Sb levels in the red blood cells of rats and observed that the Sb concentration was dose-dependent and higher in female rats. Further experiments [254,287,288,289,290,291,292], while presenting somewhat different results, corroborate that there is at least a degree of absorption.

Despite ample evidence from animal trials, the results cannot be easily correlated to humans due to some significant constraints, outlined in [144]: the administered forms contain much more Sb than would happen in realistic conditions; the chemical forms of the ingested Sb are not frequently found in nature; and in all organisms, other dietary and health factors affect absorption and tolerance of antimony.

### 4.7. Effects on the Skin

What few data exist on Sb and skin interaction originate from studies on smelter workers and miners. The first such research was performed by the authors of [293], who recorded skin irritations. The so-called ‘antimony spots’, i.e., antimony-associated skin lesions, were recorded in [294]. Antimony exposure has also been linked to occupational dermatitis by some researchers [295,296]. In any case, the appearance of antimony spots is considered a rare occurrence [262]. From a histological perspective, these lesions exhibit necrosis and acute inflammation closely related to the sweat ducts [294]. The lesions, which often exhibit eczema and lichenification, resemble those of smallpox; they occur in many body locations, except for the feet, hands, and face [262].

An interesting curative use of antimony was its use in Mohs paste [297], which was developed in the 1930s by F.E. Mohs [298], who observed that a 20% solution of zinc chloride caused cellular death but preserved the general histological structure [299]. The application of this paste, albeit with an altered composition, is the basic step of the still-in-use, fresh-tissue Mohs chemosurgery technique, proposed in the 1960s, to rectify the increased incidence of recurrence of basal cell carcinomas after the initial surgery had taken place [300].

The Mohs chemosurgery was found to be highly effective in the case of basal cell carcinoma [300] as well as other rarer cases, such as the excision of cylindromas [301]. The advantages of zinc chloride, the basic component of Mohs cream, are that it is a good fixative and its permeation can be controlled through the application of a paste of specific composition [302]. The stibnite served as the granular part and *sanguinaria canadensis*, commonly known as bloodroot powder, served as the powder. It is worth noting that Mohs tested a number of compounds as in situ fixatives, including antimony trichoride, which, however, distorted tissue structures [303]. Even so, the application of the paste was painful and sometimes caused lymphadenopathy; local inflammation and fever were also not uncommon [303]. Such effects are more associated with the toxicity of bloodroot, whose other preparations have similar or even more adverse side-effects [304]. Such problems were obviated with the introduction of the fresh-tissue technique, where no paste is applied. The current iteration of the paste, which has a number of applications, as discussed below, uses neither stibnite nor bloodroot.

The clinical application of the cream began in 1936, following successful in vivo trials in rats; the initial applications of the method were highly successful [305,306,307,308]. Gradually the fresh-tissue Mohs chemotherapy was developed which led to ever more efficient tumor excisions (e.g., [309,310,311,312]). It must be noted, however, that the high success rates of the technique are attributable to the fixative and not the anti-cancer properties of the paste [313], as demonstrated by [314]. Of course, the process also has some drawbacks, in that it was painful and time-consuming and the devitalization of the tissues made the closure of the incision difficult [315]. Today, fresh-tissue micrographic surgery, which does not use the paste, is much more rapid and less discomforting to the patients.

Apart from chemotherapy, the Mohs paste has proven useful in a number of occasions and clinical settings (e.g., [315,316,317,318,319]), although stibnite is no longer used to provide the granular part; rather the current composition of the paste comprises zinc chloride, distilled water, zinc starch, and glycerol [320].

## 5. Use of Antimony in Medicine

While, as mentioned before, the uses of antimony can be traced back to ancient civilizations, it was Paracelsus who promoted its use during the 17th century in Europe. However, the systematic use of antimony in modern medicine can be attributed to Plimmer and Thomson, who used it to treat African trypanosomiasis [321]. Soon, the side effects of antimonial drugs, which include but are not limited to headache, nausea, vomiting, diarrhea, ache in the muscles and joints, coughing and syncopes, and anaphylaxis, became apparent [322]. Interestingly, there is a single, apparently positive report, in using antimonials to treat syphilis [323]. Some attempts were also made to use Sb against malaria [324,325] and at least one attempt was made to treat framboesia tropica (non-venereal endemic syphilis) [326]. Currently, the use of antimony in the treatment of lung tumor cell lines [120] is being studied. In addition, quite recently, the use of Sb dithiocarbamate complexes has been studied for their potential antibacterial activity, with promising results [327], and they have also exhibited a noteworthy antifungal activity [328]. Based on the general anticarcinogenic principle of action of dithiocarbamate compounds, Sb-dithiocarbamate compounds can be considered as potential anticarcinogenic agents [329]. The anticarcinogenic potential of Sb has already been mentioned in relation to the research of [120].

A summary of the most relevant applications of antimony in medicine is presented in Table 4.

### 5.1. Antimonial Drugs for Leishmaniasis Treatment

Leishmaniasis can be regarded as a complex zoonosis and is caused by protozoans of the genus *Leishmania*. About 20 species of *Leishmania* are infectious to humans, and their vectors are different species of female phlebotome sandflies [350,351]. Currently, it is considered endemic in Africa and Asia, but even in Western countries, it is a problem in HIV-infected patients [352], or patients who are otherwise immunosuppressed for medical reasons. The partial or even complete lack of a functional immune system leads to increased parasite burdens and compromised treatment response [353]; such patients are more liable to develop antimonial drug resistance [354,355]. According to [352], patients with concomitant AIDS and leishmaniasis can infect sandflies which will further spread the disease; the same is not true for immunocompetent patients. This emerging problem can be partially mitigated by the use of anti-retroviral drugs, a course of treatment that is, however, not universally available [356].

The most common use of antimonial drugs in medicine concerns in the treatment of leishmaniasis. There exist two main types: trivalent antimonials, also known as tartar emetics, and pentavalent antimonials.

The first confirmation of antimonial drug efficacy was provided in [357] against cutaneous leishmaniasis, and in [358,359] against visceral leishmaniasis. Despite the initial hopeful results, the toxicity of the drugs soon became evident; a problem additionally compounded by their apparent instability in tropical climates [360], where the disease is most prevalent. Other reports, however, refs. [361,362] indicated that the use of tartar emetics was ineffective to the point that it was proposed that, perhaps, no treatment would be preferable. A symptom of cutaneous leishmaniasis are the so called ‘oriental sores’ and reports of Sb use in their treatment is mentioned in [363,364].

Nowadays, the classic therapy for leishmaniasis is pentavalent antimony, to which there appears to be, however, increasing resistance. In cases of such an occurrence, liposomal amphotericin B is preferred, which is, however, much more expensive. It has been observed, by various researchers over the years [365,366,367,368,369,370] that trivalent Sb compounds are toxic to both stages of *Leishmania* parasites, i.e., the amastigotes occurring with the mammals and the promastigotes occurring in the sandflies; by comparison, pentavalent antimonials are only toxic to amastigotes.

### 5.2. Antimonial Drugs for Human African Trypanosomiasis Treatment

Human African trypanosomiasis is a serious condition, which will prove fatal if left untreated. The number of worldwide reported cases is remarkably low, but its regional distribution and localization are pronounced in sub-Saharan Africa. The pathogenic species responsible for the disease are the unicellular protozoans *Trypanosoma brucei gambiense*, and *Trypanosoma brucei rhodesiense*; while *Trypanosoma brucei brucei* infects only animals. Tsetse flies of the *Glossina* genus are responsible for the transmission of Trypanosomes [371]. The pathogenic *Trypanosoma* species are immune to the actions of the human physiological trypanosomal lytic factor [372].

The choice of drugs for treating human trypanosomiasis is rather limited. For the first stage of the disease caused by the *T.b. gambiense*, pentamidine is used, either intramuscularly or intravenously [373,374]. For the intermediate stage of the disease, pentamidine has proven rather ineffective.

Being a tropical disease, antimony was once a considered a prime candidate as a potentially curative agent [336]. Today the use of antimonials is sparse, owing to their very high toxicity, both in cases of humans, and when used to treat animal trypanosomiasis [375]; despite that, in vitro results [338] and in vivo studies [337,339] of some antimonials have proved positive.

### 5.3. Antimonial Drugs for Schistosomiasis Treatment

Schistosomiasis, also known as bilharziasis, is the second most prevalent tropical disease after malaria [376], and is common in tropical and subtropical regions [377,378,379]. Schistosomiasis is caused by worms of the genus *Schistosoma* [379], with water snails being the intermediate hosts of the parasite. Schistosomiasis can be distinguished between acute, also known as Katayama fever, and chronic [376].

Drugs based on antimony inhibit glycolysis and other metabolic pathways [362]. In 1918, sodium antimony tartrate began being used for the treatment of schistosomiasis [340,341] and it was found to be quite effective until the 1960s [380,381,382] under different treatment protocols. Different antimony drugs, or in different doses, were also used in [342,343]. An attempt in [344] to use an oral antimony salt had disappointing results and it was concluded that trivalent sodium antimony tri-gluconate was ineffective when administered orally. Comparative trials of different antimonials demonstrated, in the case of urinary schistosomiasis at least, that the most effective drugs were accompanied by the most severe side effects [345]. A new chemical form of the standard antimony sodium tartrate was proposed in [346] in the early 1970s, and according to an experiment conducted by the researchers, it was characterized by better tolerance. A few years before, in 1968, a very successful use of sodium antimony tartrate was reported in [347], for patients specifically infected with *S. haematobium*.

Today, antimony drugs are no longer used to treat schistosomiasis, mainly because of their cumulative toxicity and the fact that the maximally active Sb(III) linked to oxygen species was very toxic, while the less-toxic Sb(III) linked to sulfur species was also less active. Other antimonial drug formulations were attempted [348,349], but towards the 1970s, the use of Sb in treating schistosomiasis was abandoned.

From an early stage, special consideration was given to the adverse effects of antimonials to the myocardium. Initially, there were some reports of death shortly after [383] or sometime later [384], following the administration of trivalent antimony compounds. Further researchers also noted anomalies in cardiograms of treated patients and heart-related pathologies ([264] and references therein). Based on these reports, the authors of [264] conducted research on the electrocardiograms of the treated patients, reporting slight changes in most patients, while in a number of patients the changes were so severe as to indicate myocardial disease induced by the treatment. While the degree of the changes could not be correlated with the dose, in each individual they became progressively worse during the course of the treatment. The most prominent changes were in the T wave, and in all cases they diminished fairly rapidly after the cessation of the treatment [264].

### 5.4. Resistance to Antimonial Drugs

The initial use of antimonial drugs for over half a century did not indicate any notable development of resistance [321], although, perhaps, such observations are difficult to make, given the regional variation of treatment protocols. For example, in most areas of South America, Africa, and Asia, where the disease is prevalent, the standard treatment protocol is a dose of 20 mg per kg each day of a pentavalent antimonial, which is administered parenterally, for a period of about a month. In the Mediterranean region, the treatment of choice is liposomal amphotericin B (L-AmB). Both of the treatments mentioned involve immunocompetent patients [108].

The cardinal rule behind drug resistance is that a drug with the smallest ratio of half-life to therapeutic efficacy has the lowest possible chance of inducing resistance. This explains, for example, the very high efficacy of amphotericin B deoxycholate in the case of leishmaniasis [385,386].

The resistance to antimonials is most probably associated with the detoxification mechanism of the cells, as mentioned above. In the particular case of leishmaniasis, but in other tropical diseases too, the choice of drug depends on efficacy, toxicity, cost, and availability, in that order of importance [387].

It is not currently known if the inducement of resistance to antimonials can be attributed to their extensive use or to their specific properties in terms of their absorption and action inside the human body.

## 6. Discussion and Conclusions

As is apparent from the section on antimony in the food chain, most foodstuffs are considered safe, although the relevant studies are rather limited, and frequently the detection methods employed are not sensitive enough to detect Sb, even though its presence is speculated. Hence, the confidence level of analytical studies remains relatively low [144].

According to the literature review in [388] there is a potential for micronutrients to modulate the adverse effects of heavy metal intoxication, and hence antimony intoxication. More specifically, dietary sufficiency or insufficiency can greatly modulate the risk assessment of such metals.

Taking into account the rising public concern regarding the accumulation of prospective harmful elements and other contaminants, both in the geosphere and biosphere [389], detailed investigations into Sb pollution and contamination are required. Some case studies for some pollutant loads exist for rivers (e.g., [390,391]) and other ecological sites.

In the specific context of soil contamination assessment, the geological background should always be taken into account [392]; a holistic ecosystem approach, as proposed by [393] might be optimal for this purpose.

Further research is required in the future to ascertain the dispersion of Sb, via the atmosphere in urban settings. This is further illustrated by the link between airborne particles and disease in such settings (e.g., [394,395]). Research along the lines of the methodology of [396] would be useful in such an endeavor.

## Figures and Tables

**Table 1:** Industrial uses of antimony compounds.

Antimony Compound	Chemical Formula	Uses
Antimony trioxide	Sb_2_O_3_	Flame retardant in plastics, textiles and rubber; catalyst for PET production
Antimony pentoxide	Sb_2_O_5_	Flame retardant
Sodium antimonate	NaSbO_3_	Flame retardant; decolorizing and refining agent for optical glass
Antimony trisulfide	Sb_2_S_3_	Photoconductors, brake linings, fireworks
Antimony pentasulfide	Sb_2_S_5_	Vulcanizing agent
Antimony triacetate	Sb(CH_3_COOH)_3_	Catalyst in the production of polyesters

**Table 2:** Economically important antimony minerals.

Mineral	Chemical Formula	Crystal System	Mineral Group	Color	References
Stibnite	Sb_2_O_3_	Orthorhombic	Sulfides	Gray with luster	[39,40]
Jamesonite	Pb_4_FeSb_6_S_14_	Monoclinic	Sulfosalts	Gray to black	[41,42]
Valentinite	Sb_2_O_3_	Orthorhombic	Oxides	White to light grey to yellow	[43,44]
Senarmonite	Sb_2_O_3_	Cubic (Isometric)	Oxides	Colorless to grey	[45,46]
Stibiconite	(Sb^3+^Sb^5+^)_2_O_6_(OH)	Cubic (Isometric)	Oxides	White, yellow, orange to light brown	[47,48]
Bindheimite	Pb_2_Sb_2_O_6_O	Cubic (Isometric)	Oxides	Yellow to brown to greenish brown	[49,50]
Kermesite	Sb_2_S_2_O	Triclinic	Sulfides	Red	[51,52,53]
Tetrahedrite	Cu_6_(Cu_4_C_2_^2+^)Sb_4_S_12_S	Cubic (Isometric)	Sulfosalts	Various shades of grey	[54,55]

**Table 3:** Soil and water Sb pollution in selected Sb mining sites.

Location	Region, Country	Sb Water Content (μg/lt)	Sb Soil Content (mg/kg)	References
Ouche	Massif Central, France	200–350	n/a	[108]
Pernek	Malacky, Slovakia	1–31	121–894	[109]
Dúbrava	Žilina, Slovakia	4–9300	4.8–9619	
Medzibrod	Banská Bystrica, Slovakia	11–1290	2–793	
Poproč	Košice, Slovakia	5–1000	13–6786	
Čučma	Košice, Slovakia	1–3540	6.2–782	
Su Sergiu	Sardinia, Italy	23–1700	19–4400	[110]
Glendinning	Dumfries & Galloway, Scotland	0.10–783	6.77–261	[111]
Endeavour Inlet	New Zealand	14.1–30.4	18–243	[112]
Llorenç d’Hortons (industrial site)	Barcelona, Spain	1.93–2.06	0.1–112	[113]
Losacio-Las Cogollas	Zamora, Spain	n/a	60–230	[114]
Bardo	Lower Silesia, Poland	0.14–0.76	n/a	[118]
Bystrzyca Górna		0.13–123	n/a	
Czarnów		0.01–16.6	n/a	
Dębowina		0.33–437	n/a	
Dziećmorowice		0.05–151	n/a	
Srebrna Góra		0.02–170	n/a	
Puqing mining area	Guizhou, China	n/a	0.49–1431	[106]
Huangshi	Hubei, China	n/a	0.62–4.65	
Xikuangshan	Hunan, China	n/a	100–5045	
Keramos	Chios Island, Greece	115.94–478.63	n/a	[119]

**Table 4:** Summary of medical uses for antimony compounds.

Pathology	Compound and Administration	Dosage	Pathogenic Factors Targeted	Application	References
Cancer	Trivalent antimony potassium tartrate	4.2–322 µg/mL	small cell lung cancer cell lines	in vitro (currently under research)	[330]
Syphilis	Antimony powder in saline solution—intravenous injections	50–200 mg	*Treponema pallidum*	in vivo (historical use)	[323]
Malaria	Various	Various	*Plasmodium* spp.	in vivo (historical use)	[324,325,331]
Framboesia tropica	Antimonium tartarum—intramuscular	Various	*Treponema pallidum pertenue*	in vivo (historical use)	[326,332]
Various bacterial infections	Sb(ephedtc)_3_ and monophenylantimony(III) compounds—microtiter plates & salt application	21.4–125.6 µM	*P. aeruginosa*; *E. coli*; *K. pneumoniae*; *Salmonella dublin*; *E. cloacae*; *S. aureus*; *E. caseofluvialis*; *S. sciuri*; plus multiresistant clinic isolated strains	in vitro (currently under research)	[327,328]
Aspergillosis	Monophenylantimony(III) compounds—Salt application	27.9–65.08 µM	*A. niger*; *A. flavus*	in vitro (currently under research)	[328]
Leishmaniasis	Sodium antimony gluconate; meglumine antimoniate—intramuscular	10–100 mg/kg	*Leishmania* spp.	in vivo	[321,333,334,335]
Trypanosomiasis	Various combinations of antimonials and other compounds	Various	*Trypanosoma* spp.	in vitro (experiments in murine trypanosomiasis); in vivo	[336,337,338,339]
Schistosomiasis	Various antimonials—intravenously, intramuscular	3.5–530 mg	*Schistosoma* spp.	in vivo (historical use)	[340,341,342,343,344,345,346,347,348,349]

ephedtc = ephedrinedithiocarbamate ligand.

## Data Availability

Not applicable.
